# The influence of corporate market power on health: exploring the structure-conduct-performance model from a public health perspective

**DOI:** 10.1186/s12992-021-00688-2

**Published:** 2021-04-06

**Authors:** Benjamin Wood, Owain Williams, Phil Baker, Vijaya Nagarajan, Gary Sacks

**Affiliations:** 1 Global Obesity Centre, Deakin University, Geelong, Australia; 2 School of Political Science and International Studies, University of Leeds, Leeds, UK; 3 Institute for Physical Activity and Nutrition, Deakin University, Geelong, Australia; 4 Macquarie Law School, Macquarie University, Sydney, Australia

**Keywords:** Market power, Corporate power, Industry structure, Firm conduct, Market failure, Antitrust policy

## Abstract

**Background:**

The detrimental impact of dominant corporations active in health-harming commodity industries is well recognised. However, to date, existing analyses of the ways in which corporations influence health have paid limited attention to corporate market power. Accordingly, the public health implications of concentrated market structures, the use of anti-competitive market strategies, and the ways in which market power mediates the allocation and distribution of resources via market systems, remain relatively unexplored. To address this gap, this paper aimed to identify and explore key literature that could inform a comprehensive framework to examine corporate market power from a public health perspective. The ultra-processed food (UPF) industry was used to provide illustrative examples.

**Methods:**

A scoping review of a diverse range of literature, including Industrial Organization, welfare economics, global political economy and antitrust policy, was conducted to identify important concepts and metrics that could be drawn upon within the field of public health to understand and explore market power. The Structure-Conduct-Performance (SCP) model, a guiding principle of antitrust policy and the regulation of market power, was used as an organising framework.

**Results:**

We described each of the components of the traditional SCP model and how they have historically been used to assess market power through examining the interrelations between the structure of industries and markets, the conduct of dominant firms, and the overall ability of markets and firms to efficiently allocate and distribute the scarce resources.

**Conclusion:**

We argue that the SCP model is well-placed to broaden public health research into the ways in which corporations influence health. In addition, the development of a comprehensive framework based on the key findings of this paper could help the public health community to better engage with a set of policy and regulatory tools that have the potential to curb the concentration of corporate power for the betterment of population health.

**Supplementary Information:**

The online version contains supplementary material available at 10.1186/s12992-021-00688-2.

## Background

The public health community has for decades identified the risks associated with the strategies and practices used by corporations that profit from health-harming commodities [1–7]. Recently, public health scholars in the emerging field of the *corporate and commercial determinants of health* (CDoH) have called for increased scrutiny of the power of corporations active in health-harming industries and its influence on population health [8–12]. Indeed, it is extensive power that confers corporations with the ability to shape many different aspects of society. Corporate power is present in socio-political aspects such as policy-making processes; in the supply chains and retail environments that structure product affordability, accessibility and availability; and in the consumption habits of individuals around the world [13]. To date, however, the public health literature has mainly explored corporate power in terms of its impact on population health via its political, regulatory and governance influence [14]. While analyses that focus on how corporations influence policies and regulations are undoubtedly important, we argue that insufficient attention has been devoted to understanding and examining the *market* power of corporations as a determinant of health, and indeed as a key explanation and source of corporate political power.

In market economies, the market system is the institutional context wherein most economic activities, notably the allocation of resources and profit accumulation, are coordinated [15–17]. Understanding who gets what and why, and at what cost, as mediated by market systems requires an understanding of market power [18]. Market power, like the concept of power in general, is a contested subject. In this paper, we draw upon Stiglitz’ (2017) description of market power as the ability of firms to engender anti-competitive market conditions through conduct that prevents, lessens or distorts the structure and process of competition [19]. The primary goal of shaping competition can be understood as creating anti-competitive market conditions conducive to generating sustained profit margins over what would otherwise be possible in a truly competitive market [19, 20]. This, in turn, can grant the ability to accumulate substantial wealth and resources over a sustained period of time, and precedes and reinforces the expression of political power by corporations. Indeed, the ability to influence politics and policy generally requires the accumulation and effective deployment of large amounts of money and other material resources [21, 22].

We argue that the omission of market power from public health analyses has led to a number of key disciplinary blind spots. First, minimal acknowledgement has been given to examining and understanding important market structure characteristics that confer firms with structural power vis-à-vis consumers, buyers, suppliers, small rival firms, and, in some cases, governments. Monopolistic/oligopolistic (i.e. market structures with only one or a small number of firms) and monopsonistic/oligopsonistic (i.e. a market dominated by only one or a small number of purchasers of given goods) market structures are of particular concern given that they often provide leading firms with both the ability to control the production, marketing and distribution of products in the value chains in which they operate, as well as the power to generate profits at the expense of other market stakeholders, including consumers and suppliers [19, 23]. Indeed, the majority of health-harming industries are oligopolistic in nature (and, in some cases, oligopsonistic), which has important ramifications for efforts to curb the consumption of the products they produce, market, and sell [23, 24]. Furthermore, concentrated markets often confer dominant firms with considerable structural and relational power relative to governments, not least through their ability to control large amounts of capital and labour. This structural and relational power can increase government hesitancy to implement policies and regulations that could threaten the profit-making abilities of these dominant firms, as well as the wider stability and health of national economies [25, 26].

Second, limited public health attention has been given to analysing the allocation and distribution of resources, generated wealth and incurred costs via the market systems of key health-harming industries [27]. The presence of considerable market power in private markets can lead to the inefficient allocation and distribution of resources, wealth, and costs – this constituting a key component of what is commonly referred to as ‘market failure’ [28]. Concentrated market power can also be harnessed to influence democratic political processes, leading to situations – such as when government policy makers and regulatory bodies are co-opted to serve private interests instead of public interests – in which many of the social and ecological costs associated with business activities (i.e. negative externalities) are not addressed [29, 30]. Accordingly, from a public health perspective, market power analyses can inform an examination into how much profit a health-harming corporation generates; how much of this accumulated capital is subsequently allocated to corporate strategies known to undermine public health; and how the generated wealth and incurred costs (including externalised costs) from market transactions are distributed among corporations, society at large, and the environment.

Finally, market power analysis is well-placed to help the public health community engage with a broader set of government policy levers that could promote and protect public health through addressing concentrated market power and related instances of market failure [31, 32]. Pertinent examples include policy regimes (e.g. antitrust policy, trade and investment policies, industrial policy) that (in principle) directly address concentrated market power, as well as fiscal policy regimes intended to redistribute the economic burden of externalised costs from the public towards the corporations responsible for driving the costs.

Given the aforementioned blind spots and opportunities, the aim of this study was to identify and explore key literature that could inform the development of a comprehensive framework to examine corporate market power from a public health perspective. We focused on the ultra-processed food (UPF) industry (refer to Supplementary File 1 for a definition) to provide illustrative examples. The UPF industry was selected for two main reasons: i) the UPF industry is a key industry of public health concern, given that diets characterised by high UPF consumption have been linked with a range of adverse population health outcomes, including all-cause mortality [2, 3, 33–55]; and ii) the markets of the UPF industry are markedly heterogeneous across both product and geographic boundaries, and therefore the industry is well-placed to highlight the importance of a comprehensive approach to examine market power [56, 57].

## Methods

### Literature review

A scoping review of a range of literature was performed to identify relevant concepts and approaches to identify and monitor corporate market power. The initial search strategy involved a review of scholarly articles, books and other relevant documents in public health, business, economic, political economy, and antitrust policy literature. These were sourced from Scopus, Web of Science, Medline, Business Source Complete, ABI Inform, Passport, Thomas Reuters Westlaw and Lexis Advance databases. The key search terms used were market power, monopoly/oligopoly power, monopsony/oligopsony power, and market failure. The initial search was supplemented by snowball searching in order to find further documents in the academic and grey literature. We did not set any date limits for the search. We included documents that were published in English and that presented market power concepts and approaches in accordance with the analytical framework (discussed below) that we thought could inform an examination of market power from a public health perspective. In addition, we included documents that described potential areas of government policy for countering corporate market power. Where relevant, we identified illustrative examples of market power with regard to the UPF industry.

### Analytical framework

**Fig. 1:**
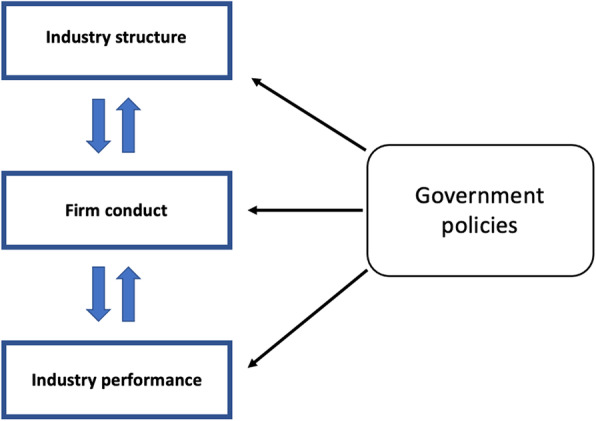
The Structure-Conduct-Performance Model, based on Carlton and Perloff (2000) [58]

We used the Structure-Conduct-Performance (SCP) model as an organising framework for our analysis (see Fig. 1) [58].

The SCP model was first described by Edward Mason and Joe Bain in the 1930s, and later popularised by Bain in the 1950s, as part of a broader agenda to identify and address both the market power and political power of firms active in concentrated markets through government policy, particularly antitrust policy [19, 59–62]. Since its introduction, it has been very influential in Industrial Organization and antitrust scholarship, fields that have traditionally concerned themselves with the structure and functioning of industries and their markets [63]. The traditional SCP approach posited that it was industry structure (i.e. the number and concentration of firms active in a given market) that determines firm conduct (i.e. firm strategy and behaviour) within the industry. In turn, firm conduct was seen to determine industry performance (i.e. the efficiency of an industry in using, allocating and distributing resources, as well as its degree of innovation) [60, 63, 64]. Given its deterministic nature, the analytical and methodological centrepiece of the traditional SCP approach was the analysis of industry structure, with a major emphasis on the use of sectoral or market concentration indices [63]. Accordingly, a central argument made by traditional SCP scholars was that the government should intervene to regulate concentrated markets, positing that doing so would prevent anti-competitive firm conduct from occurring, thereby averting the inefficient allocation of resources [58].

Detractors of this structuralist approach were critical of its deterministic nature, arguing that firm conduct (e.g. the pursuit of mergers and acquisitions) could also shape industry structure, and that firm performance shapes firm conduct (e.g. firms with substantial accumulated earnings can deliberately incur short-term losses to drive out competitors, or innovative firms could leap frog market leaders) [65]. New approaches were proposed, including those by the New Empirical Industrial Organization scholars, which placed greater emphasis on exploring profits (i.e. the financial gain realised by a firm when earnings exceed the total amount of expenditure) and price mark-ups (i.e. the difference between the cost of a good and service and its total cost of production) to analyse the extent of market power [66]. However, these new approaches also faced criticism due to their methodological and empirical challenges and limitations. As a pertinent example, the data required to accurately calculate price mark-ups are often very difficult, if not impossible, for researchers to obtain in the absence of insider information [20, 65]. Today, a number of antitrust (competition) authorities around the world instead draw from an adapted SCP model (as represented in Fig. 1), in which the relationships between each of the components are not assumed to be one directional and deterministic, and where market interventions by the government can potentially target all three components [58, 67–69]. We used this adapted model to frame our analysis.

### Data synthesis

The data extracted from the literature were coded and grouped according to the three interlinked components of the SCP model: industry structure, firm conduct, and industry and firm performance. Within each component, key concepts and related metrics for analysis of corporate power from a public health perspective were identified and discussed. A detailed description of these metrics is provided in Supplementary File 2. A glossary of key market power-related terms and definitions are provided in Table 1.

**Table 1:** A glossary of key terms and definitions related to market power

Allocative efficiency	The allocation of society’s limited resources to their most valuable use
Barriers to entry (market)	Market-based structural factors that impede or prevent a new firm from entering a market
Brand/product differentiation	The ability of a firm to differentiate its brands/products from those of its rivals
Common shareholder ownership	The situation that arises when investors, usually institutional investors, own shares in a number of firms active in the same market
Distributive efficiency	The distribution of costs (including externalised costs) and benefits generated from market transactions in the fairest and most just manner
Dynamic efficiency	The ongoing development of both process innovations (e.g. improvements in organisational, production or delivery methods that reduce cost or increase quality) and product innovations (i.e. new product and packaging development) that provide benefits to both the firm and society at large
Gross profit margin	The difference between the total sales revenue and the total cost of production
Industry	A group of establishments that are engaged in the same or similar kinds of production activity
Intangible assets	An asset, such as a brand or trademark, that does not have a physical or financial embodiment
Market	A market is the product and geographic space in which rivalry and competition take place
Market capitalisation	The discounting of a corporation’s expected, risk-adjusted future profit and interest payments to their present value
Market failure	The situation defined by an inefficient allocation and distribution of resources, wealth and costs in a market system.
Market strategy	A concerted pattern of actions taken in the market environment for the purpose of improving corporate performance (i.e. maximising profits and shareholder returns)
Monopoly	A market structure in which only one firm sells a particular commodity
Monopsony	A market structure in which there is only one firm that buys goods and services offered by many suppliers
Non-market strategy	A set of actions designed to improve or protect overall corporate performance by influencing the interconnected policy, regulatory, institutional, ideological and broader socio-political structures that shape market environments
Oligopoly	A market structure in which only a few firms sell a particular commodity
Oligopsony	A market structure in which there are only a few firms that buys goods and services offered by many suppliers
Productive efficiency	The production of products or services at the lowest possible cost
Share repurchase	The practice of a firm buying back its own shares
Transfer pricing	The manipulation of pricing and payments for intermediate outputs, brand names and patent use between subsidiaries in order to maximise profits in low-tax jurisdictions
Vertical integration	The extent to which a firm owns or controls its suppliers, distributors or buyers

## Results

### The structure of an industry and its markets

An assessment of industry and market structure is a crucial step in exploring the market power of firms, and there are a number of metrics that can be used to help identify and determine the extent of structural market power that certain firms hold [60, 64, 67, 70].

#### Market concentration

For decades, market concentration has been central to market structure analysis [60, 71, 72]. Highly concentrated markets, often understood as both a symptom and cause of market power, have long been recognised to confer dominant firms with a considerable structural advantage over other market-based actors, including consumers, suppliers, and new market entrants [60, 71–73]. The two most common metrics to measure market concentration are the Herfindahl-Hirschman Index (HHI) and the four-firm concentration ratio (CR4) [74]. The HHI, calculated by first squaring the market shares (as percentages) of all active firms in the market and then summing all of these together, is the main market concentration metric used by competition authorities today [75]. The higher the calculated value (maximum of 10,000), the greater the concentration of the market. The European Central Bank (and the United States Department of Justice prior to 2010) considers markets with a HHI value of greater than 1800 to be highly concentrated; between 1000 and 1800 to be moderately concentrated; and less than 1000 to be of a low concentration [76, 77].

In comparison, the CR4 – the most relevant concentration metric prior to the development of the HHI – is calculated by summing the market shares of the largest four firms in the market. A market with a CR4 of greater than 60 is often considered to be a concentrated market; whereas a market with a CR4 of less than 40 is often considered to be competitive [74, 78]. In most cases, the HHI is generally preferred to the CR4 because, unlike the CR4, the HHI takes into account all firms active in the market, as well as large variations in the market shares of the top firms [74]. As an illustration, consider two hypothetical markets, Market A and Market B. In Market A, the top four firms have market shares of 65, 10, 3, and 2%, respectively. In Market B, the top four firms all have market shares of 20%. Let us also assume that the remaining share of both markets is evenly spread among a large number of firms, meaning that their market shares are minimal and therefore do no impact our calculations. The CR4s of both Market A and Market B would be 80 (highly concentrated), despite their markedly different market structures. In comparison, the HHI of Market A would be close to 4338 (very highly concentrated) whereas the HHI of market B would only be around 1600 (moderately concentrated). In this case, the large difference between HHI values is due to the fact that Market A, unlike Market B, is dominated by only one firm.

A major challenge of measuring market concentration is being able to accurately define the product and geographic boundaries of the market in question [79, 80]. In this respect, it is important to point out that although the terms ‘industry’ and ‘market’ are often used interchangeably, they are not the same concepts. According to the Organisation of Economic Co-operation and Development (OECD), industry is loosely defined as a group of establishments that are engaged in the same or similar kinds of production activity [81]. A market, in comparison, is the product and geographic space in which rivalry and competition take place [82]. The UPF industry, as an example, can be described as a group of firms engaged in the production of UPFs. A number of different product markets exist within this industry because competition does not take place between all UPF product types, nor does it occur across all geographic boundaries. For instance, at the consumer level, breakfast cereal manufacturers are not in direct competition with soft drink manufacturers; therefore, we can assume that these are separate markets of the UPF industry. Likewise, a firm that only sells breakfast cereals in China is not in direct competition with a firm that only sells breakfast cereals in Western Europe. Table 2 provides an illustration of the differences in HHI values according to how market boundaries are defined.

**Table 2:** An illustration of the variation in Herfindahl-Hirschman Index (HHI) values within the ultra-processed food industry according to the defined product and geographic boundaries of the market

	Soft drink market	Carbonated soft drink market
**Global**	586	2661
**Western Europe**	562	2932
**United Kingdom**	818	3493

Note: HHI values > 2500 = very high concentration; 1800–2499 = high concentration; 1000–1799 = moderate concentration; < 999 = low concentration. Thresholds based on adapted European Central Bank and historical US Department of Justice Thresholds [76, 77]

Source: Passport. Market share data based on 2019 off-trade sales data. Product market boundaries based on Passport’s categorisation of soft drink products. The soft drink market includes carbonated soft drinks, juice, concentrates, ready-to-drink tea, ready-to-drink coffee, energy drinks, sports drinks, Asian specialty drinks, and bottled water. Carbonated soft drinks include cola carbonates and non-cola carbonates (e.g. lemonade, ginger ale)

As can be seen in the Table 2, one of the potential risks in defining the product and geographic boundaries of UPF markets too broadly is that the calculated market concentration values may underestimate the true concentration of the market in question. For instance, if we calculated the market concentration of the entire global UPF market, we could be misled into thinking that the market is competitive (because of the apparent low concentration). In reality, however, the global UPF market is made up of many different, albeit often interconnected, oligopolistic and oligopsonistic market structures (this is often captured in the economic literature by the somewhat oxymoronic term monopolistic competition) [23, 83]. Indeed, in many cases, dominant global corporations are located at the apex of a patchwork pyramid of product and geographic markets in which their market power is variable, but nonetheless typically concentrated in the majority of their key markets. Therefore, the structural market power of a global firm that operates in a number of UPF markets across different product and geographic dimensions is best explored by considering the firm’s presence in each of the markets in which it operates – across both product and sometimes segmented market-geographic dimensions – rather than by simply assessing its share of the entire global market.

#### Common shareholder ownership and its effect on market structure

The potential effect of common shareholder ownership on the competitive structure of a market or industry is an important consideration that can be incorporated into market concentration calculations. Common shareholder ownership refers to the situation wherein investors, usually institutional investors, own shares in a number of firms active in the same market [84, 85]. Evidence suggests that common shareholder ownership can be detrimental to competition, especially in highly concentrated markets, due to increased management incentives to either tacitly or explicitly collude with rivals [84, 86–88].

Recent work has highlighted the extent of common ownership across a number of key sectors in the global food value chain, including the UPF manufacturing sector [85]. Five key institutional investors (Blackrock, Vanguard, State Street, Capital Group, and Fidelity) together own between 15 and 25% of large global food corporations Mondelez, Tyson Foods, Kellogg, PepsiCo, General Mills, The Coca-Cola Company, and Dr. Pepper [85]. To capture the effect of common shareholder ownership, the modified HHI (MHHI) metric is commonly used [84]. The MHHI, which can be interpreted in the same manner as the HHI, effectively translates the estimated degree of control or influence by common shareholders over firms in the same market into a market concentration value [84]. The MHHI is determined by estimating the anti-competitive effects of common ownership (referred to as the MHHI delta), and then adding this value to the original HHI value of the market. The steps required to calculate the MHHI delta are detailed elsewhere [89].

#### Barriers to entry

An examination of barriers to market entry – anti-competitive practices and structural factors that can impede or prevent a new firm from entering a market – is another important component of market structure analysis. The presence of considerable barriers to entry confer incumbent firms with structural market power by reducing the countervailing threat of potential competition [90]. One of the major barriers to entry in many UPF markets is the presence of considerable product and brand differentiation, referring to the ability of a firm to differentiate its products and brands from those of its rivals [23]. As a result of many years of sustained and extensive marketing, large firms that have been active for a long period of time typically have a substantial advantage over new firms in that they are already well-recognised and enjoy a large and loyal consumer base. A case in point is the Coca-Cola Company and its flagship brand Coca-Cola, which, for decades, has been one of the most recognisable brands in the world [91]. Other cost-based market barriers to entry include the productive economies of scale that have been achieved by large and often globalised incumbent firms, as well as their well-established relationships and networks with suppliers, distributors, and large retailers [23, 72, 82, 90, 92–98]. Moreover, the nature of the products in many industries, such as the UPF industry, means that there is rarely substantial innovation that would break down established market-based barriers to entry.

#### Other elements of market structure

A number of other important market structural elements also warrant examination in market power analysis. For instance, vertical integration – the extent to which a firm owns or controls its suppliers, distributors or buyers – can confer a firm with a considerable structural advantage over its rivals or new market entrants [99]. Additionally, the degree of import penetration (i.e. the extent to which domestic consumption comes from imports), the export share of production (i.e. indicates the importance of foreign markets for a given industry), and the existence and nature of explicit or tacit collusion also affect the competitive structure of an industry [72, 82, 100].

Lastly, the globalisation of production is important in exploring certain structural advantages that firms may have over other competitors, as well as governments [25]. For instance, large firms with subsidiaries in different countries can readily mobilise capital across borders, thereby enabling them to undertake practices designed to minimise tax obligations and maximise financial returns [101–103]. Transfer pricing – the manipulation of pricing and payments for intermediate outputs, brand names and patent use between subsidiaries in order to maximise profits in low-tax jurisdictions – is one such example [104, 105]. A core financing strategy of Nestlé, for instance, has been described as locating its trademarks and patents in Switzerland, its home jurisdiction, in order to set up transnational intra-firm royalty payments designed to repatriate profits in tax-effective ways [102]. Similarly, the US Tax Court recently judged that between 2007 and 2009 The Coca-Cola Co had illegally transferred its profits to low-tax jurisdictions in order to avoid about 9 billion USD in income tax obligations [106].

### Firm conduct

Firm conduct is often understood through the analysis of firm strategy, which is well-placed to explore the ways in which corporate power is exercised and distributed in a variety of interconnected contexts, including market systems [8–11].

#### Market strategy analysis

In the business literature, firm strategies are often divided into two components: market strategy and non-market strategy [107]. Firm market strategies can be defined as concerted patterns of actions taken in the market environment for the purpose of improving corporate performance (i.e. maximising profits and shareholder returns) [107].

Traditionally, early SCP scholars paid particular attention to the conduct of firms active in highly concentrated markets, such as price gouging and collusion [65]. Later, this structure-centric approach to market strategy was adapted and somewhat inverted by Michael Porter, the founder of modern market strategy, who effectively argued that corporate managers would be well-placed to maintain and increase firm profitability by understanding the competitive structure of their industry [107–109]. Specifically, Porter’s five framework – one of most well-known strategic management frameworks – provides a useful analytical framework to understand how dominant firms deploy market strategy to maximise profits over a sustained period of time, largely through achieving and maintaining market dominance (refer to Supplementary File 3 for a brief description of Porter’s five forces framework) [108, 109]. Adapting this model to the UPF industry, Wood et al. (2021) describe how dominant UPF corporations use a range of market strategies to maintain market structural advantages over competitors, new market entrants, suppliers, retailers and consumers (e.g. acquire rival firms or new start-up companies with promising technologies; raise barriers to entry through extensive brand differentiation and supply chain control strategies; shape retail environments through practices such as exclusive dealing arrangements) [99]. In this respect, the boundaries between firm conduct analysis and market structure analysis are clearly blurred. A key objective of market strategy for dominant firms is to maintain market dominance through shaping the structure of the markets in which they operate. In turn, this can confer dominant firms with an increased ability to influence the behaviour of other market-based actors, thereby further consolidating their market dominance and ability to maximise profits over a sustained period of time. Such a perspective also highlights the somewhat ambiguous nature of differentiating between the deployment of effective market strategies, at least from the firm’s perspective, and the potential need for government intervention to address anti-competitive conduct [110].

#### An integrated approach to corporate strategy

Markets do not exist in a political vacuum, and firms also deploy a large range of non-market strategies, defined as a set of actions designed to improve or protect overall corporate performance by influencing the interconnected policy, regulatory, institutional, ideological and broader socio-political structures that shape market environments [9, 107, 111–114].

Differentiating market and non-market strategy can be heuristically useful to explore how power imbalances are created or exacerbated in either market or non-market environments. However, in reality, market and non-market strategies often work in tandem – captured by the term Integrated Strategy – and, in many circumstances, the distinction between these two components of corporate strategy is artificial [107]. This artificial separation between market and non-market dimensions of corporate strategy is especially apparent and important to recognise in market economies wherein neoliberal-driven structural and ideological changes have further entrenched markets and market thinking into social and political structures, if not as a cultural meta-phenomenon [115, 116]. Take, for example, Nestlé’s strategy to control marketing channels outside of the conventional market environment (e.g. healthcare facilities) as a means of increasing the revenue it generates from breast milk substitute products [117]. This strategy, well-known to pharmaceutical corporations, effectively entails the incorporation of healthcare actors (e.g. nurses, doctors, midwives and community health workers) into the firm’s marketing channels [117, 118]. As a broader illustration, the corporate social responsibility strategies deployed by large UPF manufacturers are often considered to have both a market-strategy dimension (e.g. by increasing brand value) and a non-market strategy dimension (e.g. through gaining political and consumer legitimacy) [119].

### Market and firm performance

Examining the performance of an industry entails an assessment of the use, allocation and distribution of resources and wealth that occurs via its markets [120, 121]. Initially, the performance component of the SCP approach focused on determining the extent to which monopolists and oligopolists misallocated resources through raising prices above the marginal cost of production [65, 122]. From the 1970s onwards, however, the focus of industry and market analysis shifted towards consumer welfare, largely as a consequence of the emerging dominance of the so-called Chicago School with regard to what is now a much diluted antitrust policy, led by prominent scholars such as Judge Bork [120, 123–126]. Bork and his colleagues promoted the idea, which is still widely accepted today, that consumer welfare could best be understood as consumer surplus (i.e. low consumer prices) [120, 123–126]. This relatively narrow view of consumer welfare has major problems from a public health perspective. First, the idea that low consumer prices are the key signal of the efficiency and effectiveness of the allocative and economic welfare function of private markets means that minimal regard continues to be given to the broader social and ecological outcomes of industry and market activities [127, 128]. Relatedly, and of fundamental importance, the focus on low consumer prices has served to legitimise concentrated corporate power on the grounds that it promotes economic efficiency, both in terms of driving down prices, as well as in creating related efficiencies associated with concentrated market power (we discuss some of these below) [31, 129].

Thus, in light of these considerations, there remains a clear need for the field of public health to outline a broader and more holistic approach to examine industry performance. We argue that this can, at least in part, be achieved by incorporating a broader range of social and ecological considerations into the assessment of economic efficiencies, especially allocative and distributive efficiencies [120, 121].

#### Market power and allocative (in)efficiency

Allocative efficiency refers to the allocation of society’s limited resources to their most valuable use [69]. Importantly, allocative efficiency can only be achieved if the market in question is fair and competitive (i.e. when all players in a market operate on a level playing field). The presence of considerable market power by one or more firms often results in allocative inefficiency because firms with substantial market power can generate profits – at the expense of consumers and other market stakeholders – in excess of what would be possible in a competitive market environment [19, 130, 131]. In this respect, a key aspect of market power analysis is to examine the financial performance of dominant firms, which in turn can provide insight into the degree of allocative inefficiency of the market in which they operate.

There exist a range of performance-based metrics that, when linked with firm conduct and market structure analyses, can inform an examination of the extent of market power held by a firm. Long term trends of market capitalisation values – defined as the discounting of expected, risk-adjusted future profit and interest payments to their present value – is a pertinent example, and, for publicly listed companies, can be calculated by multiplying the number of shares a firm has by its share price [15]. In the critical political economy literature, market capitalisation has been described as a ‘*symbolic ritual that converts and reduces qualitatively different power relationships into a singular quantity*’ [15]. In effect, market capitalisation speaks not just to market power, but more broadly to corporate power, given that the capitalisation process essentially quantifies the social, political and economic influence of a corporation [132].

Related to market capitalisation is the ability of a firm to generate future earnings from its assets and liabilities. In general, the larger the earnings of firm, the greater the capitalisation [15]. Examining the earnings of a firm can complement the assessment of a firm’s market capitalisation, and in cases where market capitalisation cannot be determined (e.g. unlisted firms), earnings can instead be used to roughly estimate the ability of a firm to capitalise its differential power relations [15]. A commonly used indicator of corporate earnings is ‘Earnings before Interest, Tax, Depreciation and Amortisation’ (EBITDA) – a metric that captures a firm’s earnings prior to financial and accounting deductions [133]. Similar to market capitalisation, for market power analysis, an examination of a firm’s earnings is best conducted over a long period of time (e.g. over 10 years), because firms with substantial accumulated profits can often deploy strategies that incur short term financial losses to drive out rivals (e.g. using predatory pricing) [65].

Gross profit margins, calculated by subtracting the total cost of production from total sales revenue, is another informative firm performance metric for market power analysis [134]. Although gross profit margins are less reliable as an indicator of market power compared to metrics such as the Lerner Index (i.e. (consumer price of a product – marginal cost of a product)/consumer price of a product), the latter requires data that are often not accessible. Gross profit margins tend to be most revealing in cases where the gross profit margins of a firm are considerably higher than its competitors, as well as the industry and sector averages, over a sustained period of time [135].

The value of intangible assets owned by a firm should also be considered when identifying and monitoring market power. An intangible asset (e.g. brand loyalty, patents, trademarks) is an asset that does not have a physical or financial embodiment [136]. It has been described that in the current global political economy, power has tended to accumulate into the hands of a limited number of firms that own the majority of intangible assets relative to what exists within the global value chain they operate [137, 138]. This metric is particularly pertinent for the UPF industry given that brands – aspects of which can be trademarked – play a crucial revenue-generating role for large UPF manufacturers [139–141].

#### Other forms of allocative inefficiency

Allocative inefficiency also results from other market-based failings, all of which are deeply interlinked and perpetuated by market power. A highly important category of allocative inefficiency at the firm level occurs when firms fail to incorporate *all* of the costs of a product or service into the price set by the relevant market. Externalised costs – commonly referred to as negative externalities – essentially occur when firms are not held financially accountable for the collateral damage they cause to society and the environment [69, 142]. Although not typically framed in this way, the CDoH literature contains a vast amount of evidence that describes, and, in some cases, quantifies, a diverse range of social and ecological-related negative externalities generated by a number of health-harming industries, including the UPF industry [2, 32, 143, 144]. Examples include, among many, the substantial global disease burden from diets high in UPFs, as well as the devastating ecological impact of the plastic pollution generated by UPF manufacturers [2, 3, 33–55, 145–147]. Similarly, the CDoH literature has also explored the ways in which the corporate allocation of money and resources towards certain practices, such as lobbying and intense marketing of unhealthy products, exacerbates the externalisation process [9, 11, 27, 148, 149].

Allocative inefficiency also occurs when information relevant to consumer purchasing decisions, as well as other market transactions, is not evenly shared and made readily available. This type of market failure is often referred to as the presence of information asymmetry [130]. There is ample evidence that highlights that information asymmetry is commonplace in UPF markets, where practices such as misleading labelling and the failure to disclose all relevant social (including health) and environmental costs are frequently adopted by large firms [19, 99, 130].

#### Distribution of costs and benefits

All forms of allocative inefficiency in private markets are typically compounded by an unequal distribution of costs (including those externalised) and benefits generated from market transactions [150]. As an illustrative example, the externalised social and ecological costs (e.g. increase in global burden of non-communicable diseases; the adverse ecological impact from plastic pollution) created by the global soft drink industry disproportionately affect particular demographic groups, social classes, and geographies (e.g. young children; lower socioeconomic classes; low- and middle-income countries) [32, 151–160]. On the other hand, shareholders and corporate executives – groups that tend to be over-represented by a small and privileged elite – largely benefit from the wealth generated from the soft drink industry [19, 161, 162]. Therefore, we argue that an examination of the distribution of incurred costs, as well as the corporate transfer of wealth, should be incorporated into a critical performance analysis from a public health perspective, and more broadly, into an expanded economic concept of consumer or social welfare. Corporate wealth transfer mechanisms that warrant scrutiny include the extent of tax minimisation, the value of dividends paid to shareholders relative to the wealth transferred to other stakeholders (e.g. employees), and the value of shares repurchased by a firm [163]. Share repurchases refer to the practice of a firm buying back its own shares, which has been described as a way of effectively redistributing wealth to shareholders and company executives through the manipulation of share prices and performance metrics (e.g. earnings per share) linked to executive pay compensation [164, 165].

#### Dynamic efficiency

Dynamic efficiency refers to the development of both process innovations (e.g. improvements in organisational, production or delivery methods that reduce cost or increase quality) and product innovations (i.e. new product and packaging development) that provide benefits to both the firm and society at large [166]. There is conflicting evidence as to whether market power increases or decreases innovation [167, 168]. In any case, in relation to health-harming industries, product and process innovations that address the negative externalities of pre-existing processes and products should, of course, be welcomed. However, innovations that pretend, or only partly address, pre-existing externalities should be scrutinised, and if appropriate, discredited. It has been described, for instance, that many innovations of the UPF industry adopt nutritional reductionist and greenwashing principles, and thereby fail to provide true social and ecological benefits [169, 170].

#### Productive efficiency

Productive efficiency refers to the production of products or services at the lowest possible cost [121, 123]. In certain situations, such as when firms have achieved economies of scale in production, market power and productive efficiency can have a direct relationship [166]. High levels of productive efficiency of health-harming industries pose a clear problem for public health because, in the majority of markets, the lower the consumer price of a health-harming product, the greater its consumption, and thus, the greater its public health burden. Given the often perverse relationship between lower consumer prices and greater public health costs, the public health community should concern itself with comparing the productive efficiency between health-harming corporations and the organisations that produce healthier and more sustainable product alternatives.

#### Use of natural resources

Finally, an assessment of economic efficiencies from both a public and planetary health perspective should also take into account that ecological resources are finite, and that issues pertaining to the scale of resource use cannot simply be reduced to issues of allocation or distribution [150]. Several authors argue that, in many cases, it may not be appropriate for the use of scarce natural resources (e.g. land, water) for business activities, as well as the production of waste and pollution that results from business activities (e.g. carbon emissions, plastic pollution), to be coordinated by the price signals of a market [150, 171, 172]. Instead, it is argued that social decisions that reflect ecological limits need to be made to organise resource use [150]. In this respect, the public and planetary health implications of a firm’s relationship with key planetary boundaries (e.g. climate change, freshwater use, land use, biodiversity loss, and chemical pollution), as described by Rockström et al. (2009) and popularised by Raworth’s Doughnut Economics model, could be considered as a separate, albeit interlinked, source of economic efficiency [171, 173]. As an example, in certain water-scarce regions of the world, concerns have been raised about the ways in which the bottlers of Coca-Cola Co and PepsiCo’s soft drink products have exacerbated the rapid depletion of water sources for surrounding communities [174,175].

### Towards the development of a framework to examine market power from a public health perspective

**Fig. 2:**
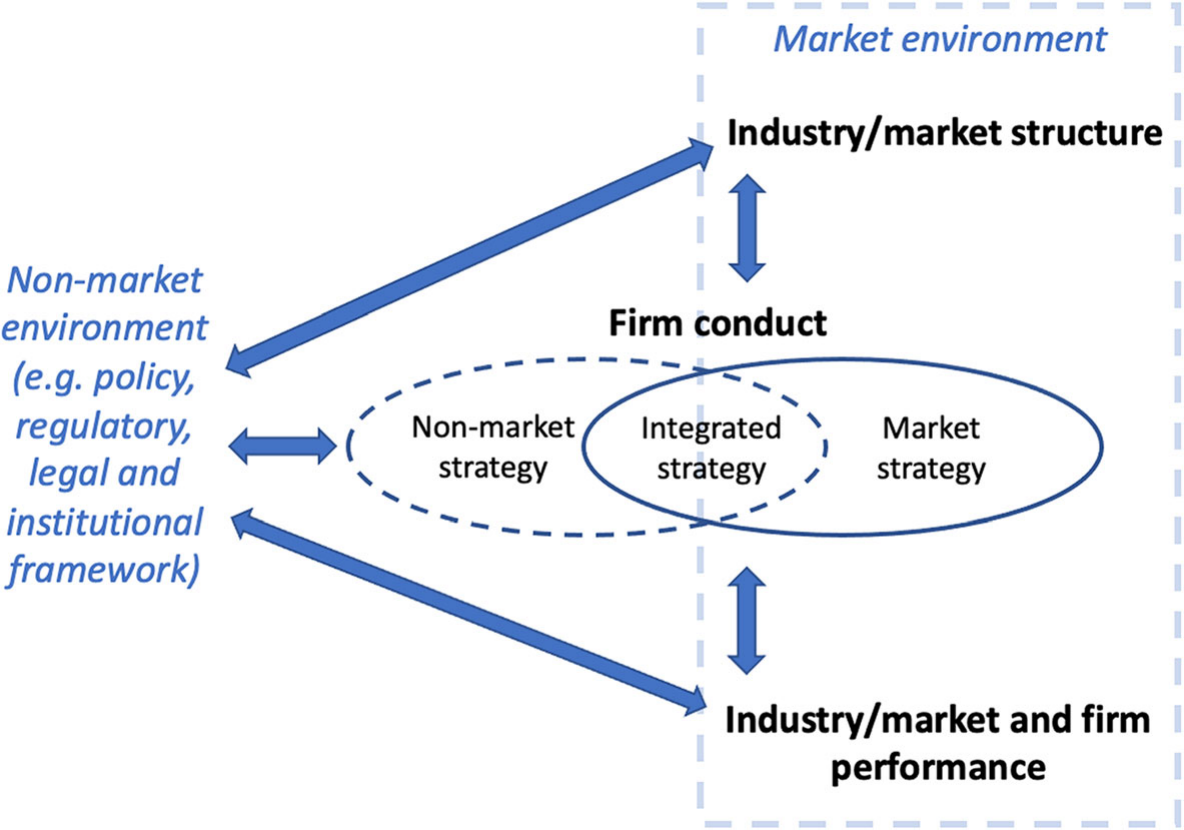
An adapted SCP model that could be used to inform the development of a comprehensive framework to analyse market power from a public health perspective

Drawing from our findings, we argue that an expanded SCP model described throughout the results section and depicted below (refer to Fig. 2) could serve as a useful point of departure to inform the development of a comprehensive framework to analyse market power from a public health perspective. Such a framework would be well-placed to explore the ways in which market power mediates, and indeed is mediated by, industry and market structure, firm conduct, and market and firm performance. Moreover, by comprehensively exploring market power through the interconnected analysis of the three components of the SCP model, the public health community could be better equipped to identify and engage with key government policy regimes that could be used to curb concentrated market power.

#### Locating market power within the broader non-market environment

We recognise that market systems are embedded within the underlying political, legal, cultural, ideological, and technological contexts of capitalist societies, which are systems that markets and capital have historically co-produced [17]. In this respect, market power research needs to be linked with work that explores the existence, use and distribution of corporate power in social and political structures traditionally outside the reach of markets and market thinking [11]. For example, market power analysis could be complemented with an exploration of corporate influence in and on the underlying structures and networks that govern and regulate the rules, processes and norms of the markets in which they operate [176]. Tools for such endeavours include network analysis, and closer monitoring of industry interactions with government bodies, committees, and regulators.

From a theoretical perspective, and in similar fashion to Mikler (2018), we conceputalise corporate market power and corporate political power as a perpetuating unity, rather than as separate entities [177]. Corporate market power can lead to accumulated material resources that can create and reinforce corporate influence in the political environment (i.e. political power), just as corporate political power can both protect (e.g. through regulatory capture) and perpetuate corporate market power (e.g. through shaping corporate-friendly trade and investment agreements) [11]. In addition, the existence, use and distribution of corporate power varies across time and space, and can be fungible across and between markets and jurisdictions. For instance, the increased material power of a firm that results from its power in a specific market can be later deployed to influence the political environment in a different jurisdictional space [11].

#### Examples of government policy to address concentrated market power

The proposed framework is well-placed to help explore a number of policy levers that can protect and promote the interests of public health through curbing market power. It is beyond the scope of this paper to review all such relevant policy regimes; instead, we briefly discuss the potential role of a few key examples.

Antitrust policy is a policy regime that, we argue, deserves more attention from public health scholars. Under most current antitrust policy frameworks around the world, the scope for government intervention into markets through antitrust enforcement is limited. As mentioned earlier, this is largely due to the ideological hegemony of the Chicago School’s thinking on antitrust policy, wherein concentrated market structures and consolidated market power are justified and legitimised on the grounds of consumer surplus arguments [31, 178]. Historically, however, the primary goals of antitrust policy were to prevent and address the concentration of market power as means of both promoting economic justice and protecting democracy [19, 30, 31, 61, 179]. Today, the neo-Brandeisian anti-monopoly movement (named after Justice Brandeis who served under President Wilson in the early 1900s) seeks to revive the historical goals of antitrust policy, and it is with this movement that public health could aim to engage [180]. Although the main focus of this movement is currently on the big digital technology corporations (in particular, Google, Facebook, Amazon, Apple, and Microsoft), there is likely to be merit in the public health community participating in antitrust policy discussions with reference to addressing (other) health-harming oligopolies that drive the CDoH [180].

Beyond the way in which the goals of antitrust policy are interpreted today, another important limitation of current antitrust policy regimes around the world is that they are typically bound by their jurisdictional boundaries, and thus, in most cases, fail to operate across geographic boundaries (and more broadly, planetary boundaries) [171, 181]. This is a major issue given the globalised nature of value chains and market transactions, as well as the fact that the social and ecological burden of certain business activities clearly transcend jurisdictional boundaries [171]. In this respect, trade and investment policies could serve as an important lever for addressing the market power of global corporations, especially those that operate in health-harming industries and seek to expand their reach and dominance into new developing markets [19]. A challenge for low- and middle-income countries (LMICs) in particular, however, is that neoliberal global trade, investment and intellectual property regimes often facilitate the globalisation and concentration of market power of firms based in advanced capitalist economies, especially the US and the EU and its members [19]. International trade agreements, for instance, often restrict the ability of LMICs to prevent powerful, health-harming foreign corporations from entering and penetrating their developing national economies [19].

As a final example, industrial policy could also serve as an important policy lever to address the market power of dominant health-harming corporations. Specific to the food sector, industrial policy – that is, government policy that shifts resources from one industry to another – could play a critical role in promoting the economic efficiency of organisations that produce healthier and more sustainable food alternatives to those of UPF manufacturers [182]. Policy actions in this respect could encompass government investment in infrastructure that supports local food supply chains for perishable products (e.g. improved transport infrastructure and cold chains); supporting farmers to engage in direct sales in produce markets; increasing support for alternative food business models such as food cooperatives; developing integrated food and agricultural knowledge and innovation systems to address information asymmetries inherent in the food and agricultural value chain; and implementing policies that mandate the public procurement of healthy, local and sustainable food products [183–185].

## Discussion

In this paper we have used the SCP model to identify and describe key concepts and metrics that could inform the future development of a comprehensive framework to analyse corporate market power from a public health perspective. We have provided a number of examples throughout the paper specific to the UPF industry. Building upon the work of scholars who have, for decades, attempted to identify and monitor the presence, use and outcomes of market power, we have described how the SCP model is well-placed to help public health researchers explore the interrelations between the structure of industries, the conduct of dominant firms within these industries, and the overall ability of markets and firms to efficiently allocate and distribute the scarce resources of society. Throughout the paper, we have also suggested how the SCP model could be expanded to take into account a broader range of public health considerations.

While our paper outlines key concepts and metrics to guide market power analysis, it will be necessary to develop a detailed protocol for analysis, including the identification of available data sources, specification of time frames for analysis, and guidance for interpretation of all indicators and metrics. Once developed, such a framework could be adapted to examine market power in different industries of public health concern, such as UPFs, fossil fuels, alcohol, tobacco, and gambling. Regarding the UPF industry, a comprehensive market power framework could be incorporated into the work of existing research networks interested in examining the role of the private sector in shaping and influencing food supply chains and food environments. INFORMAS (International Network for Food and Obesity/non-communicable diseases Research, Monitoring and Action Support) is one such example, and research on market power could be well-placed to help achieve the aims of the network [186]. Given the general concerns about how corporate wealth transfer mechanisms contribute to wealth and social inequality, a comprehensive market power framework could also help inform an exploration of the ways in which market power can exacerbate wealth and social inequalities, especially in market economies [122, 164].

A key strength of this paper is that it links work from economics, business, global political economy and antitrust policy literature with public health. Tackling a complex topic such as market power demands such an interdisciplinary approach. The paper has a number of important limitations. While we have attempted to provide a comprehensive set of concepts and metrics for analysis, we recognise that some of the data required for such analysis is not publicly accessible. While certain data can be collected from publicly available sources such as annual firm reports, national trade databases, and stock exchange websites, access to some of the data (e.g. historical company fundamental data, sales revenue data disaggregated by product and geography) typically requires subscriptions to a number of market and business-related databases. Furthermore, there are currently no standardised benchmarks to guide interpretation of many of the key metrics discussed. In any case, contextual analysis is important and interpretation needs to consider a range of findings. Nevertheless, benchmarks are likely to emerge over time and this could assist future analyses. Finally, a key challenge of the framework described in this paper is that public health practitioners may not have the necessary skills in economics, business, finance and antitrust policy to use and interpret some the proposed metrics. Building these skills should therefore be considered a focus area, particularly for those scholars interested in scrutinising and addressing corporate market power. Fostering collaborations with groups from economics, finance, business and antitrust policy would also help in this regard.

## Conclusions

There is increasing evidence that the unchecked corporate power of dominant firms, especially those in health-harming commodity industries, poses a major threat to efforts to protect and promote population health. Accordingly, it is critical that the public health community focuses on understanding, identifying, tracking, and addressing corporate power.

Corporate market power is a vital power concept that warrants more public health attention as part of a broader corporate power research agenda. We argue that market power can be broadly understood as playing a central role in perpetuating corporate power through the shaping of market environments as a means of ensuring the ongoing accumulation of substantial profits. Empirically, the SCP model described in this paper could act as a useful starting point to examine corporate market power from a public health point of view, as well as to engage with the policy, regulatory and institutional levers designed, at least in principle, to curb the concentrated market power of firms that generate profit at the expense of population health.

## Supplementary Information

**Additional file 1: Supplementary file 1.** Definition of the ultra-processed food (UPF) industry

**Additional file 2: Supplementary file 2.** A number of key structure and performance-related metrics to examine the market power from a public health perspective

**Additional file 3: Supplementary file 3.** A brief description of Porter’s five forces framework

## Data Availability

Not applicable.
