# Supplementary material for: The influence of corporate market power on health: exploring the structure-conduct-performance model from a public health perspective
Source: Global Health. 2021 Apr 6;17:41. doi: 10.1186/s12992-021-00688-2 (PMC8025506; doi:10.1186/s12992-021-00688-2)
Supplement: Supplementary file 1 — Additional file 1: Supplementary file 1. Definition of the ultra-processed food (UPF) industry [file 12992_2021_688_MOESM1_ESM.docx]

**Supplementary file 1. Definition of the ultra-processed food (UPF) industry**

We used the food classification system NOVA to define UPFs [1]. The NOVA system categorises food products into four groups according to their level of processing [2, 3]. The first group consists of unprocessed and minimally processed foods. The second group includes processed culinary ingredients, such as oils, butter, sugar, lard, and salt. The third group consists of processed foods made by adding salt, oil, sugar or other substances from the second group to foods from the first group. The fourth and final group are UPFs, which include food products such as soft drinks, confectionery, sweet biscuits, ice-cream, and savoury snacks [4, 5]. UPFs are made from combining substances derived from foods with synthetic additives via a series of industrial techniques and processes [1].

We considered the UPF industry to consist of firms whose primary business activity is to manufacture UPF products. Examples of large UPF manufacturing corporations include Nestlé, The Coca-Cola Company, PepsiCo, Danone, Mars, Unilever, Mondelez International, and the Kraft-Heinz Company. Retailers of UPF products (e.g. supermarkets), as well as suppliers of inputs for UPF production (e.g. sugar producers), were considered to be part of the UPF value chain, but not part of the UPF industry itself.

**References**

1. Monteiro C, Cannon G, Lawrence M, da Costa Louzada M, Machado P. Ultra-processed foods, diet quality, and health using the NOVA classification system. Rome: Food and Agriculture Organization of the United Nations; 2019.

2. Moubarac J-C, Parra DC, Cannon G, Monteiro C. Food Classification Systems Based on Food Processing: Significance and Implications for Policies and Actions: A Systematic Literature Review and Assessment. Current Obesity Reports. 2014;3:256-72.

3. Baker P, Machado P, Santos T, Sievert K, Backholer K, Hadjikakou M, et al. Ultra-processed foods and the nutrition transition: Global, regional and national trends, food systems transformations and political economy drivers. Obes Rev. 2020.

4. Monteiro C, Cannon G, Lawrence M, da Costa Louzada M, Machada PP. Ultra-processed foods, diet quality, and health using the NOVA classification system. Rome: Food and Agriculture Organization of the United Nations; 2019.

5. Monteiro CA, Cannon G, Moubarac JC, Levy RB, Louzada MLC, Jaime PC. The UN Decade of Nutrition, the NOVA food classification and the trouble with ultra-processing. Public Health Nutr. 2018;21(1):5-17.
