# Supplementary material for: The influence of corporate market power on health: exploring the structure-conduct-performance model from a public health perspective
Source: Global Health. 2021 Apr 6;17:41. doi: 10.1186/s12992-021-00688-2 (PMC8025506; doi:10.1186/s12992-021-00688-2)
Supplement: Supplementary file 2 — Additional file 2: Supplementary file 2. A number of key structure and performance-related metrics to examine the market power from a public health perspective [file 12992_2021_688_MOESM2_ESM.docx]

**Supplementary file 2. A number of key structure and performance-related metrics to examine the market power from a public health perspective.**

| *Important examples of metrics for industry/market structure analysis* | | | |
| --- | --- | --- | --- |
| **Indicator** | **Data required** | **Description** | **Rationale** |
| Market concentration | Sales data (disaggregated by product type and geography) | Market concentration is often measured using one of two indicators: Herfindahl Hirschman Index (HHI) and top-four firm concentration ratio (CR4). The HHI is calculated by summing the squares of all the market shares of each firm in the industry [1]. The CR4 is calculated by summing the market shares of the largest four firms in the market [2]. | HHI and CR4 are the two most commonly used indices for measuring market concentration, an essential metric in assessing market power dynamics. HHI is generally preferred for reasons described in the paper. |
| Common shareholder ownership | Ownership and sales data | Defined as the common ownership of firms in the same market by large financial investors. The modified HHI (MHHI) is a metric that estimates the potential effect of common ownership. The steps required to calculate the MHHI delta are detailed elsewhere [3]. | Common shareholder ownership can undermine competition and is therefore an important market power dynamic [4-7]. |
| Brand differentiation and equity | Brand, marketing and advertising data | Brand differentiation is a key component of product differentiation – the process of differentiating a product from competitors. Brand equity is the value premium that a firm generates from a brand when compared to a generic equivalent. | Brand differentiation and equity are important examples of barriers to market entry, especially in UPF markets [8]. |
| Other barriers to entry | Data from market research databases | Narrative review of relevant market research reports revealed other key barriers to entry, such as: i) high capital intensity ratio (e.g. high cost of processing equipment, export licences); ii) reliance on economies of scale; iii) well-established relationships with suppliers, distributors and retailers | Other metrics can support a broader assessment of existing barriers to entry. |
| Vertical integration | M&A transaction data | Vertical integration refers to the integration of firms from one functional level of the value chain into another. | Vertical integration is an important metric to assess market structure and the dynamics between different sectors of the value chain. |
| Import penetration | National trade data | Import penetration is calculated as follows:  Import value / (total – export + import) x 100 [9] | This is a proxy of competition from foreign markets, an important indicator of countervailing power. |
| Export share of revenue | National trade data | Export revenue / total revenue x 100 [9] | Highlights the importance of foreign markets for a given industry. |
| Degree of transnational production | Corporate structure data, sales and employment data in active countries | This indicator looks at the countries (and their tax frameworks) in which the companies controlled by the firm in question are based, as well as operating revenues and employment in different geographic markets. | Explores the globalisation of a firm from a production perspective, and can assist in exploring firm conduct such as tax minimisation, as well as a firm’s structural power vis-à-vis governments. |

| *Important examples of metrics for firm performance analysis* | | | |
| --- | --- | --- | --- |
| **Indicator** | **Data source** | **Description** | **Rationale** |
| Market capitalisation | National stock exchange websites, company fundamentals data (e.g. company financial statements) | Market capitalisation is the number of shares outstanding multiplied by share price. This can be calculated as a ratio relative to the average market capitalisation in the relevant industry or sector, or to the average of peer or comparator groups listed on the same stock exchange. | Can be understood as a ‘*symbolic ritual that converts and reduces qualitatively different power relationships into a singular quantity*’, as well as a measure of investor confidence – an important determinant of future investment [10]. |
| Earnings  (e.g. EBITDA) | Company fundamentals data | EBITDA (earnings before interest, tax, depreciation, and amortisation) is a measure of a firm’s financial performance that reflects earnings prior to the influence of accounting or financial related deductions. This can be calculated as a ratio relative to the average firm in the relevant industry or sector, or to the average of peer or comparator groups. | EBITDA is a measure of corporate performance and is particularly useful in situations where market capitalisation values are unable to be properly calculated (e.g. private or non-listed firms). |
| Gross profit margins | Company fundamentals data | The difference between total sales revenue and the total cost of production, and is often expressed as a percentage relative to total sales revenue. | Suggests the presence of market power in cases where the gross profit margins of a firm have been considerably higher than its rivals (as well as the industry/sector average) over a sustained period of time. |
| Lerner index or related measure of price mark ups | Insider information on marginal costs (Lerner Index), pricing and sales data (price elasticity) | The Lerner Index (L) is calculated by subtracting the marginal cost of a product (MC) from its price (P), and then dividing this by the price (i.e. L = (P-MC/P). A related measure, the price elasticity of demand, is calculated by dividing the change in quantity of a certain product by the change in its price, over a defined period of time and within a defined area. | Indicators that look at product mark ups, such as the Lerner Index, have become one of the most common ways of assessing the presence and extent of market power in mainstream and industrial economics. Unfortunately, the required data are often difficult to obtain. |
| Value of intangible assets owned | Company fundamentals data | A measure of a firm’s control over key assets (e.g. intellectual property such as brands). This can be calculated as a ratio relative to the average value of intangible assets owned by rival firms, or to the average of peer or comparator groups. | The ownership of intangibles is linked to the accrual of corporate power in the global economy. |
| Total expenditure on corporate practices that undermine public health | Company fundamentals data; company disclosure data (e.g. Security and Exchange Commission filings for US-based firms) | A measure of the amount that firms active in health-harming industries spend on practices such as advertising and related marketing practices, lobbying, political donations, funding or research and academic institutions, community sponsorship etc. | These practices can exacerbate allocative inefficiency (market failure) via mechanisms such as policy and regulatory capture, the shaping of ideas and public opinion, increased consumer demand for health-harming products etc. |
| Tax minimisation | Company fundamentals data | The effective tax rate (ETR) is a commonly used indicator used to inform an examination of tax minimisation. The ETR of a firm is calculated by dividing the total income tax paid by a firm by its pre-tax income over a given time period [11]. | The ETR can inform an examination of a firm’s ability to minimise tax obligations. It is important to note that data need to be jurisdiction specific if the researcher is interested in comparing the ETR to jurisdiction specific corporate tax rates. |
| Wealth transfer to shareholders and company executives | Company fundamentals data; dividend and share repurchase data | One way this can be measured is by summing the dividends paid and the value of share repurchases made by a firm, and comparing this with total revenue or capital expenditure [11]. | Dividend payments and share repurchases are two key financial mechanisms in which the wealth generated from a firm’s operations is transferred to shareholders and company executives. |

**References**

1. Weinstock DS. Using the Herfindahl Index to measure concentration. The Antitrust Bulletin. 1982:285-301.

2. Arnold R. Economics: Thomson South-Western; 2008.

3. Lambert TA, Sykuta ME. The Case for Doing Nothin About Institutional Investors’ Common Ownership of Small Stakes in Competing Firms. Virginia Law and Business Review. 2019;13.

4. OECD. Common Ownership by Institutional Investors and Its Impact on Competition. Paris: Organisation for Economic Co-operation and Development; 2017. Contract No.: DAF/COMP(2017)10.

5. Posner E, Scott Morton F, Weyl G. A Proposal to Limit the Anti-Competitive Power of Institutional Investors. Corporate Governance and Economics eJournal. 2017.

6. Elhauge E. Horizontal Shareholding. Harvard Law Review. 2016;129.

7. Azar JS, Martin; Tecu, Isabel. Anticompetitive Effects of Common Ownership. Journal of Finance. 2018;73(4).

8. Schmalensee R. Inter-Industry Studies of Structure and Performance. In: Schmalensee R, Willig R, editors. Handbook of Industrial Organization. Amsterdam: North-Holland; 1989. p. 954.

9. OECD. STAN Indicators: Collection of Calculation Formula. The Organisation for Economic Co-operation and Development; 2011.

10. Nitzan J, Bichler S. Capital as Power: A Study of Order and Creorder: Routledge; 2009.

11. Hager SB, Baines J. The Tax Advantage of Big Business: How the Structure of Corporate Taxation Fuels Concentration and Inequality. Politics & Society. 2020;48(2):275-305.
