# Supplementary material for: The influence of corporate market power on health: exploring the structure-conduct-performance model from a public health perspective
Source: Global Health. 2021 Apr 6;17:41. doi: 10.1186/s12992-021-00688-2 (PMC8025506; doi:10.1186/s12992-021-00688-2)
Supplement: Supplementary file 3 — Additional file 3: Supplementary file 3. A brief description of Porter’s five forces framework [file 12992_2021_688_MOESM3_ESM.docx]

**Supplementary file 3. A brief description of Porter’s five forces framework.**

Porter’s five forces framework outlines five ‘forces’ that shape the competitive structure of an industry [1, 2]:

1. Intensity of competition between rivals;
2. Threat of new market entrants;
3. Threat of substitute products (and technologies);
4. Bargaining power of buyers, including consumers;
5. Bargaining power of suppliers;

References

1. Porter ME. Competitive strategy: Techniques for analyzing industries and companies. New York: Free Press; 1980.

2. Porter ME. The Five Competitive Forces That Shape Strategy. Harvard Business Review. 2008:23-41.
